# Subsidized veterinary extension services may reduce antimicrobial resistance in aquaculture

**DOI:** 10.1038/s41598-023-37262-2

**Published:** 2023-06-21

**Authors:** Sophie St-Hilaire, Stephen Chi Ho Chan, Kwok Zu Lim, Brett MacKinnon, Tzu Hsuan Cheng, Ka Po Fiona Cheng, Aaron Chi Fai Leung, Sabrina Hei Yuet Lam, Vidya Bhardwaj, Olivia Sinn Kay Chan

**Affiliations:** 1 Department of Infectious Diseases and Public Health, Jockey Club College of Veterinary Medicine and Life Sciences, City University of Hong Kong, Kowloon Tong, Hong Kong SAR People’s Republic of China; 2 CityU Veterinary Diagnostic Laboratory Co. Ltd, City University of Hong Kong, Kowloon Tong, Hong Kong SAR People’s Republic of China; 3 Division of Community Medicine and Public Health Practice, School of Public Health, Li Ka Shing Faculty of Medicine, The University of Hong Kong, Hong Kong, Hong Kong SAR People’s Republic of China; 4 Present Address: Berrimah Veterinary Laboratory, Department of Industry, Tourism and Trade, Berrimah, NT Australia

**Keywords:** Antibiotics, Bacterial infection

## Abstract

Antibiotic use in aquaculture has become very controversial vis-à-vis driving antimicrobial resistance (AMR) in aquatic bacterial populations. The AMR trends in fish pathogens in Hong Kong over a four-year period suggests that providing small stakeholder farmers with free veterinary advice on fish health issues and treatments, as well as subsidized quality-assured medicines, likely reduced AMR. We observed a dramatic reduction in the proportion of bacteria resistant to oxolinic acid, oxytetracycline, and florfenicol on local aquaculture farms between 2018 and 2021. These decreases coincided with either a change in antibiotic use practices on farms (i.e. with oxytetracycline), or the reduction in the use of specific drugs (i.e. oxolinic acid and florfenicol). We did not observe a similar decline in the resistance pattern to commonly used antibiotics in human medicine in the same fish bacteria. Resistance to these products, which were unlikely to be used by the farmers in our study, was very high. Our finding suggests that both human and veterinary use of antibiotics in Hong Kong may have an influence on the AMR of bacteria in the aquatic environment.

## Introduction

Antibiotic use in aquaculture has been linked to antimicrobial resistance (AMR) issues in aquatic ecosystems^1–5^. Although the use of antibiotics in this industry is not the only factor driving AMR in aquatic bacterial populations, it likely plays a role in the global increase of antibiotic resistance. Most countries have regulations in place to limit the use of medicines in food animals^6,7^, and many have developed strategies and guidelines for the prudent use of antibiotics in different health sectors, including veterinary medicine for aquatic animals. Despite this, changing the mindset of health professionals and farmers is difficult, especially when alternatives to antibiotics are few, and often not as cost effective in controlling fish mortalities in the face of bacterial disease outbreaks. It is also difficult to enforce regulations on antibiotic use in aquaculture, and relatively easy for a layperson to purchase drugs in many places, particularly in low- and middle-income countries^6,8–10^. Many of these products are available on-line without a prescription, and can be hand-mixed on farms when the quantity of feed used is low. Further, surveillance for drug residues in fish products sold locally, especially in live fish markets, is often limited, so it is difficult to penalize individuals who do not comply with regulations on the use of these substances.

The use of antibiotics in fish does not directly negatively impact the animals, and it may help when fish are infected with a bacterial pathogen, so this is sometimes seen as a treatment option when fish are dying. Unfortunately, as researchers learn more about metaphylactic treatments in aquaculture, it is becoming apparent that delivering medication effectively to large populations of fish is complicated and requires careful execution to ensure proper dosing of the fish^11^. It is also evident that environmental leaching of top-coated drugs occurs^12,13^. Another issue that has become apparent in the last decade is the sale of fraudulent drugs, which contain lower concentrations of active compounds than stated on their labels^14^. The use of these sub-optimal pharmaceutical products results in under dosing, a practice known to increase the risk of AMR^15,16^.

In many aquaculture industries, particularly for small farm operators in low- to middle-income countries, which make up a significant portion of the aquaculture industry in Asia^17^, there are limited numbers of fish health experts to assist farmers with disease diagnostics and provide advice on appropriate mitigative strategies for control fish mortalities. In some settings, farmers rely on previous experiences or the farming community’s advice for treatment options. These suggestions are often done with minimal diagnostic work-up other than gross evaluation of dead fish. This type of diagnosis is not accurate, given that many fish diseases appear similar on gross examination of animals. Further, in some cases, those recommending the antibiotics may profit from the sale of these medications (i.e. sales representatives or private veterinarians), which may lead to promoting their use.

To address the lack of veterinary extension services for small aquaculture farm operators in Hong Kong, the veterinary college at City University of Hong Kong (CityU) established an Aquatic Veterinary Extension Service (AVES) for local fish farmers in September 2018. This service was subsidized by the Agriculture, Fisheries, and Conservation Department (AFCD) of the government of Hong Kong, SAR, through a Sustainability Fisheries Development Fund grant. The program consisted of a veterinary consultancy service, a non-profit pharmacy, as well as education outreach on fish health issues for farmers. The types of aquaculture clients who engaged with the AVES were small-scale stakeholders with either fresh-water ponds, brackish water ponds, indoor recirculating aquaculture systems with or without aquaponics, or net pen marine systems. At the time of this study the  aquaculture industry in Hong Kong produced approximately 2900 tonnes of fish annually from inland ponds, and 330 tonnes of marine net pen fish per year^18^. Prior to the establishment of the AVES at CityU, there were no aquaculture veterinary specialists to help Hong Kong fish farmers with medical treatments. To receive service farmers call a dedicated phone line for assistance and, depending on the history and clinical presentation described on the site, the veterinarians visit and work-up the case free of charge, including conducting bacteriology cultures and sensitivity analyses when a bacterial infection is suspected, making recommendations on mitigation strategies appropriate for the situation, as well as providing quality-assured medicines at cost when justified by diagnostic tests. Over the last 5 years the service has increased in popularity among the local fish farmers. The objective of this study was to evaluate the trends in AMR in fish pathogens isolated from Hong Kong fish farms by the AVES, to determine if there were any changes in the resistance profiles over time to commonly used drugs in aquaculture and to drugs used predominantly in human medicine.

## Methods

We extracted data from the results of the culture and sensitivity analyses done on all bacterial fish pathogens isolated from fish cases submitted to the Veterinary Diagnostic Laboratory at CityU between September 2018 and December 2021. The isolates originated from moribund fish collected by veterinarians as part of their AVES duties. Samples for bacterial isolation were only submitted when fish were suspected of having a bacterial disease. Kidney swabs were the preferred sample collected for culture purposes submitted to the laboratory. Different media (i.e. marine agar and Thiosulfate-Citrate-Bile Salts-Sucrose Agar when the affected fish were of salt water origin, and Trypticase Soy Agar for fresh water fish samples) were used for primary isolation and purification of pathogenic bacteria, which were subsequently identified using MALDI-TOF MS analysis as described in Theel et al.^19^. The antimicrobial sensitivity testing of all isolated pathogenic bacteria was done using the Kirby Bauer disc diffusion method^20^. Antimicrobial sensitivity testing was performed according to Clinical and Laboratory Standards Institute guidelines^21^ with breakpoints derived from Whitman and MacNair^22^. The antibiotics evaluated included the two drugs prescribed by the veterinary service, oxytetracycline and florfenicol, as well as oxolinic acid, which is licensed for use in fish in some countries, and streptomycin, lincomycin, and penicillin, which are commonly used in human medicine, but never prescribed by the aquatic veterinary extension service.

From the extracted laboratory data on bacterial antibiotic sensitivity, the proportion of fish pathogens resistant to each antibiotic of interest was calculated for each year of the study (i.e. from January to December) by dividing the number of bacterial isolates deemed resistant to the specific antibiotic by the total number of bacteria evaluated during the same time period. These proportions were expressed as percentages and depicted graphically over time. In the case of lincomycin, resistance to this lincosamide antibiotic was only assessed in Gram-positive bacteria in the first and last year of the study. Also, in 2018, we only had data on antibiotic resistance from September to December because the veterinary service was only initiated in September of 2018.

We also extracted information from the AVES database on the number of clients using the service, clinical cases seen (revisits to a farm site for the same problem were not included in this count), and the number of prescriptions given to fish farmers during the study period. These data were also summarized by year. The percent of cases treated with antibiotics was determined by dividing the annual number of antibiotic prescriptions issued by the annual number of cases. This was also described for each type of antibiotic used (i.e. oxytetracycline and florfenicol).

For comparison purposes we examined the usage (daily defined dose) of different classes of drugs in both inpatients and outpatients (delivered orally and parenterally) at hospitals in Hong Kong over the same time period as our study (2018–2021)^23^. For the aminoglycoside drugs, we summarized the use of streptomycin, amikacin, gentamicin, kanamycin, neomycin, paromomycin, and tobramycin. For the lincosamide drugs, we included oral and parenteral use of clindamycin and lincomycin. For estimating the use of the penicillins, we included daily defined doses (DDD) of phenoxymethylpenicillin, procaine penicillin, benzathine, benzathine penicillin, benzylpenicillin potassium, benzylpenicillin sodium, carbenicillin indanyl sodium, piperacillin, amoxycillin, and ampicillin. The use of the quinolone antibiotics included a few second and third generation drugs, specifically the DDD of ciprofloxacin, moxifloxacin, ofloxacin, and levofloxacin. Lastly, for the assessment of the tetracycline usage we included dosages of oxytetracycline, doxycycline, and tetracycline.

### Approval for use of animal veterinary data

The data used in this project originated from the Aquatic Animal Veterinary Service at the City University of Hong Kong. The Human Ethics Committee at City University of Hong Kong approved the use of the data for this project and the interviews with the practicing veterinarians (HU-STA-00000277). The veterinarians providing the service are licensed and regulated by the Veterinary Surgeons Board of Hong Kong. All veterinary services provided to the fish during the study period were subject to the approval of the owners of the fish farms. The farmers were aware that the data may be used for research purposes on an aggregated level. All data were reported in a manner that maintains the confidentiality of the clients. The funding agency for the veterinary service (the Agriculture, Fisheries and Conservation Department of the Government of the Hong Kong Special Administrative Region, China) also approved the manuscript for publication.

### Approval for use of human data

The data collected on the antibiotic use in hospitals in Hong Kong was publicly available https://www.chp.gov.hk/en/static/101607.html.

## Results

Over the 4-year study period there was an increase in the number of clients using the AVES and a gradual increase in number of cases seen by the veterinarians (Table 1). The proportion of cases which required antibiotics to control bacterial diseases was, initially, 24%. This number, however, declined in subsequent years and was approximately 10% of cases by 2021 (Table 1). Although the proportion of cases requiring antibiotics declined over time, the total number of prescriptions did not because the number of cases increased. Most of the fresh and saltwater pathogens detected by the veterinary team were either *Aeromonas* spp. (31%) or *Vibrio* spp. (46%). There were also a few *Streptococcus* spp. infections (4%) in both fresh and saltwater farms over the 4-year period.

**Table 1:** Summary information on clients, farm cases, bacterial sensitivity analyses conducted, and breakdown of antibiotic prescriptions issued by the CityU veterinary service between 2018 and 2021.

Year	Number of clients	Number of cases	Number of bacteria tested for resistance	Number of prescriptions issued	% of cases requiring prescriptions	% of prescriptions for oxytetracycline	% of prescriptions for florfenicol
2018*	8	21	19	5	24	20	80
2019	28	62	33	7	11	57	43
2020	50	87	29	8	9	88	13
2021	77	136	45	13	10	92	8

*Service commenced in September of 2018.

**Figure 1:**
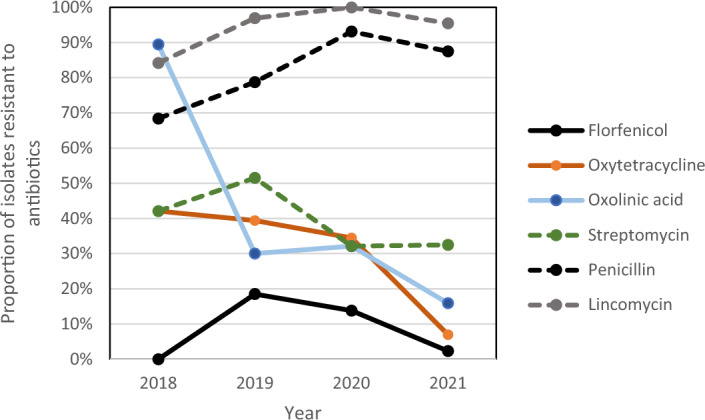
Annual percentage of fish pathogens resistant to different antibiotics between 2018 and 2021. Only the Gram-positive bacteria were assessed for lincomycin resistance in 2018 and 2021.

The OTC resistance pattern for bacteria isolated from fish over the 4-year period declined over time (Fig. 1). For florfenicol, the pattern increased between the first and second year, but subsequently declined (Fig. 1), mirroring the use of this product by the aquatic animal veterinarians (Table 1). Resistance to oxolinic acid in the pathogens tested was extremely high in the first year of the service, but rapidly declined over time (Fig. 1). The pattern of streptomycin resistance in the fish pathogens ranged between 30 and 50%, depending on the year (Fig. 1). Resistance to lincomycin appeared to increase between the first and the last year of the study (Fig. 1). The proportion of resistance to penicillin in the isolates was always very high, ranging between 70% to over 90%, depending on the year (Fig. 1).

Human antibiotic use data from hospitals in Hong Kong suggested a slight decline in the use of penicillin-like antibiotics and quinolones over the 4-year study period, and an increase in the use of tetracycline and the lincosamides over the same time period (Table 2). Based on the representative drugs that we evaluated in the publicly-available hospital antibiotic use dataset, there were considerably more penicillins, tetracyclines, and quinolones used compared to aminoglycosides and lincosamide drugs (Table 2).

**Table 2:** In and out patient use of different groups of antibiotics in Hong Kong hospitals summarized by year.

Year	Class of antibiotic				
	Aminoglycosides	Lincosamides	Penicillins	Quinolones	Tetracyclines
2018	69,707	53,731	1,076,691	1,066,574	1,028,002
2019	63,334	55,655	1,054,073	1,046,474	1,257,150
2020	61,801	54,027	873,387	925,583	1,313,283
2021	68,538	59,789	951,066	948,096	1,401,326
Total	263,381	223,202	3,955,217	3,986,727	4,999,760

The data in the table includes both oral and parenteral use^24^. Quantity was measured in “daily defined dose”. For the aminoglycoside drugs we summarized the use of streptomycin, amikacin, gentamicin, kanamycin, neomycin, paromomycin, and tobramycin. For the lincosamide drugs we included oral and parenteral use of clindamycin and lincomycin. For estimating the use of the penicillins we included daily defined doses (DDD) of phenoxymethylpenicillin, procaine penicillin, benzathine, benzathine penicillin, benzylpenicillin potassium, benzylpenicillin sodium, carbenicillin indanyl sodium, piperacillin, amoxycillin, and ampicillin. The use of the quinolone antibiotics included a few second and third generation drugs, specifically the DDD of ciprofloxacin, moxifloxacin, ofloxacin, and levofloxacin. Lastly, for the assessment of the tetracycline usage we included dosages of oxytetracycline, doxycycline and tetracycline.

## Discussion

Deciphering the role that different sources of antibiotics play on AMR in the aquatic environment has been difficult and controversial. The patterns of AMR in fish pathogens isolated from fish farms in Hong Kong provides insight into what the potential impact of using antibiotics on fish farms is on this phenomenon, and the potential role that other sources of antibiotics, unrelated to fish farming, play on AMR in the aquatic ecosystem. By evaluating the trends in resistance to antibiotics used in animals and those used predominantly in humans, we can start to see patterns that suggest both human and animal use of antibiotics influence the resistance profile of fish pathogens. Further, this study provides evidence that an aquatic veterinary extension service (AVES), which offered diagnostic services and management strategies to help reduce fish losses on small farms, and which was not dependent on the sale of pharmaceuticals for profit, may reduce AMR associated with the use of antibiotics in aquaculture.

To better understand the role that veterinary use of antibiotics has on driving AMR in fish pathogens, we studied the trend in the AMR pattern for the two drugs (florfenicol and oxytetracycline (OTC)) that the aquatic veterinary service in Hong Kong prescribed over a four-year study period. The drug doses recommended by the veterinarians for these two drugs were based on the current recommendations by the manufacturer of these products^24,25^. The pattern of resistance to florfenicol mirrored the proportion of prescriptions written for this medication during the same timeline (Table 1 and Fig. 1). This antibiotic is not used in human medicine^26^, so the AMR pattern observed is likely driven by veterinary use of the product. Although it was not possible to eliminate the role that the use of this product in the poultry and swine industries in Hong Kong may have had on the resistance pattern to this drug, these industries are relatively small in this region, and these types of farms are not in close proximity to fish farms, so discharge from these terrestrial animal farms is likely to be negligible. The florfenicol AMR pattern also suggested that the fish farmers were likely not using this drug very much before the veterinary service provided it to them as no resistance was observed in the bacteria isolated in 2018 (Fig. 1). As the veterinarians started prescribing this drug for bacterial disease outbreaks we observed an increase in the resistance to the product. The veterinarians preferred using this drug to OTC because it is not used in human medicine. It is also available in a form that is easy to mix in fish feeds and it is labeled for use in several species of fish around the world. However, despite the sensitivity profile, it is quite difficult to achieve successful treatments with this medication when the water temperatures are elevated (i.e. > 28 °C), as is commonly experienced in Hong Kong^27^. This is due to the drug’s short half-life at warm temperatures^28,29^. To avoid under-dosing fish with this product and treatment failures, the veterinarians changed their practice after the first year of the veterinary service to using OTC for treating many of their bacterial cases (Table 1) and, interestingly, the resistance to florfenicol declined over time (Fig. 1). Although this pattern could be coincidental, it may also reflect that veterinary use of drugs has an impact on endemic pathogens found on fish farms.

Florfenicol was not the only drug with an antibiotic resistance pattern that suggested the veterinary service in Hong Kong was influencing, to some extent, the AMR of fish pathogens. The reduction in the OTC AMR in fish isolates over time may reflect a more prudent use of this antibiotic by farmers under veterinary supervision. When the service started, the veterinarians observed several issues with antibiotic treatments. In the first year, farmers would call the veterinarians as a last resort when fish were already off-feed and mortality had been ongoing for some time. Antibiotic therapy when fish are off-feed results in uneven dosing and poor treatment response^11^. The veterinarians also observed farmers’ limited knowledge on how to effectively deliver medications to aquatic species. We suspect, given the practices used initially by the farming community, that sick fish may have been under-dosed and environmental contamination from uneaten medicated feed may have occurred.

In a separate study in 2018, we found several sources of OTC and florfenicol that contained lower concentrations of medications than what was reported on their labels^14^. If farmers were using products without quality assuring them, this could have also led to under-dosing of animals, which has been demonstrated to promote antibiotic resistance^15,16^. Within the first year of initiating the veterinary service in Hong Kong, the veterinarians provided quality-assured drugs to their clients, so they had accurate concentrations of the active pharmaceutical ingredient. All medications were batch tested by a local commercial chemical laboratory (Chemical Testing Services, Hong Kong Baptist University, Hong Kong) using liquid chromatography mass spectrometry^30^ to establish the drug concentration in the premixes prior to prescribing them.

Further, to improve farmers’ knowledge of AMR, training on prudent use of antibiotics, which included the importance of biomass estimations, properly top coating medicated feeds, and delivering the feed appropriately to ensure the entire population was treated, was provided in 2019. Over time, the veterinarians worked with their clients to increase knowledge on how to use antibiotics to reduce under-dosing and environmental leaching, and to improve the delivery of these drugs to their fish. Each time medications were prescribed, the veterinarians worked one-on-one with the farmers to ensure the drugs were administered as prudently as possible. The slow decline in the occurrence of OTC resistance in fish pathogens, which was approximately 40% of isolates in 2018, to less than 10% in 2021, despite the increase in the number of prescriptions for this product in the industry, suggests that the extension service may have had a positive impact on the antibiotic use practices in the aquaculture industry in Hong Kong.

The decline in resistance to OTC in fish pathogens could also have been associated with a decline in the antibiotic use in other sectors. To determine whether this was the case, we examined the use of this class of drug in local hospital populations, and did not observe any correlation with the resistance pattern in fish pathogens. In fact, the opposite was seen. There was an increase in the number of daily defined doses of tetracycline antibiotics used in hospital patients in Hong Kong over the same time period as our study (Table 2), which provides anecdotal evidence to suggest that this source of antibiotics was not driving the decline in OTC resistance in our fish pathogens. It may have influenced the baseline level of drug resistance, but we were not able to test this hypothesis with our dataset.

Another antibiotic resistance pattern observed in this study that provides further evidence of the importance of prudent use of antibiotics on farms was the large decline in resistance to oxolinic acid after 2018. This drug is a first-generation quinolone, which is approved for fish use in many countries^6,31,32^, and which is available in a form that can be easily top-coated on fish feed. The AVES veterinarians made a conscious decision not to prescribe this product and, instead, provided other antibiotics to their clients (i.e. florfenicol and OTC). Prior to the implementation of the veterinary service the use of medicines on fish farms was left up to the farmers, and there was no centralized record of prescriptions used on fish farms in Hong Kong. It is likely that antibiotics were used prior to 2018; however, we could not verify this. One explanation for the dramatic reduction in resistance to oxolinic acid in fish isolates after 2018, and the continuous decline in resistance to this drug within the 4-year study period, is that farmers stopped using this product once they received advice from veterinarians and were offered alternatives to oxolinic acid. It was considerably easier, and likely less expensive to use the subsidized drugs from the veterinary service than to purchase medications without a prescription.

It is unlikely that the human use of quinolone antibiotics drove the change in resistance to oxolinic acid in aquatic fish pathogens, as the use of these antibiotics in Hong Kong hospitals only changed slightly from 1 million DDD in 2018 to approximately 950,000 DDD in 2021, based on the three quinolones we evaluated in the hospital dataset. There was a decline in the number of fish farmed in Hong Kong in 2021 due to supply chain issues (Personal communication, anonymous farmer, 2022), which could have influenced the bacterial resistance patterns, but it could not account for the massive drop in isolates resistant to oxolinic acid between 2018 and 2019.

Unlike the patterns of resistance for antibiotics commonly used to treat fish, we did not see a similar decline in AMR towards predominantly human-use antibiotics. For example, three antibiotics which were part of the laboratory’s standard sensitivity panel, and for which the class of drugs are not available in a premix that is easy to add to fish feed (i.e. lincomycin, penicillin, and streptomycin), had relatively consistent or increasing resistance patterns over the study period. This point is important as it suggests the decline in the resistance pattern to the drugs used in fish was not a widespread phenomenon across many classes of antibiotics.

Interestingly, the proportion of bacteria that were resistant to drugs less likely to be used in fish in Hong Kong, such as streptomycin, lincomycin, and penicillin, was very high. For example, the drug with the highest level of resistance was penicillin. Depending on the year, 70 to 90% of the fish pathogens tested were resistant to this class of antibiotic. Although it is possible that farmers used penicillin to treat fish, this antibiotic is more difficult to find in a formulation that can be mixed with fish feed. Further, the AVES veterinarians did not prescribe this medication during the study period, and there were no competing veterinary services that could have prescribed these types of drugs to fish farmers during the study period. Although it was possible fish farmers were purchasing penicillin without a prescription, the AVES was providing free veterinary service and subsidized medication to a large number of the registered fish farmers in Hong Kong so it is unlikely that many were buying drugs illegally as these products would not have been subsidized. The high and persistent resistance to penicillin in pathogens isolated on fish farms in Hong Kong suggests that other sources of antibiotics, besides those used in aquaculture, were also influencing AMR in aquatic bacterial populations.

Further, this finding suggests that even if aquaculture farmers completely eliminated the use of antibiotics, the level of resistance to many of the drugs used in humans would remain very high (Fig. 1). Resistance to all three of the products that were predominantly used in human medicine, as opposed to the fish veterinary service (i.e. lincomycin, penicillin, and streptomycin), was detected in at least 30% of the isolates for all years of the study.

The high level of resistance to antibiotics used in humans was not surprising, as the region of Hong Kong has over 7 million people and 68 sewage treatment plants with varying levels of treatment^33^, so contamination of the aquatic environment is inevitable. Further, beach monitoring of *E. coli* counts by the Hong Kong Environmental Protection Agency also indicated faecal contamination of many sites close to the salt water aquaculture farms, with counts reaching higher than 500 cells per 100 ml at certain times of the year^34^. Researchers have also reported high nitrogen levels associated with sewage outflow in the region^35^, corroborating the a potential exposure to faecal waste and antibiotics^36^. Although the human use of antibiotics is no doubt much greater than the use of antibiotics by the small aquaculture industry in Hong Kong, and the level of influence of these two sources of environmental antibiotic exposure is likely proportional to their use, we believe we were able to detected a change in the resistance pattern of fish pathogens that was indicative of the use of certain products on the farms themselves.

Despite the fact that we did not have many pathogens for monitoring AMR on fish farms in Hong Kong, and we did not include pre and post treatment samples nor environmental samples to monitor the impact of our treatments, the 4-year dataset from the veterinary service provides subjective evidence that the use of antibiotics on fish farms may influence AMR in the aquatic environment. Trends in OTC resistance suggest that despite an increase in use, the prudent use of this product may positively affect AMR in fish pathogens. Over time, as more farmers used the service, the AMR profiles for both drugs used by the industry (OTC and florfenicol) declined, and this trend was not observed for other antibiotics commonly used in human medicine. The resistance to these other drugs remained quite high throughout the study period.

It is important to note that although the gradual reduction in the drug resistance to OTC and florfenicol in common fish pathogens in Hong Kong coincided with the introduction and adoption of a veterinary service, the extension service provided to the farmers had many facets, and it was not possible to attribute the findings reported in this study to any one individual activity. For example, the AVES programme worked on improving disease prevention strategies at the same time as it provided farmers with diagnostic services and treatment strategies that specifically targeted the cause(s) of their fish mortalities. The service also worked with the farmers to improve their husbandry to reduce the predisposing factors leading to bacterial infections, while treating the condition. It is important to point out that the veterinarians only prescribed antibiotics when they confirmed a bacterial disease on a farm and that they decoupled the sale of antibiotics and other pharmaceuticals from the service to eliminate any conflict of interest that might be linked to making a profit from the use of medications on farms. When non-bacterial diseases were the cause of fish mortality on farms, the veterinarians offered mitigation strategies that targeted the specific issue(s). This practice likely reduced the overall use of antibiotics, as these non-bacterial causes of death may have been treated with antibiotics in the past, when diagnostic services were limited or unavailable. The type of veterinary extension service that is described in this study takes several years to establish, as it requires farmer acceptance, participation, and trust. It was also only possible to provide this service because of a government subsidy. Without this source of funding, the small fish farm operators would not have been able to afford the service or been able to purchase high quality medication. However, if established properly, such a service may be a partial solution to reducing AMR attributable to the use of antimicrobials on fish farms.

## Data Availability

The datasets analysed in the current study are not publicly available due to the sensitive nature of the information and the confidentiality agreement under which the data were obtained; however, they may be made available from the corresponding author on reasonable request with assurances that the information on farmers will remain anonymous.

## References

[1] Cabello FC, Godfrey HP, Buschmann AH, Dölz HJ (2016). Aquaculture as yet another environmental gateway to the development and globalisation of antimicrobial resistance. Lancet Infect. Dis..

[2] Watts JEM, Schreier HJ, Lanska L, Hale MS (2017). The rising tide of antimicrobial resistance in aquaculture: sources, sinks and solutions. Mar. Drugs.

[3] Topp E, Larsson DGJ, Miller DN, Van den Eede C, Virta MPJ (2018). Antimicrobial resistance and the environment: assessment of advances, gaps and recommendations for agriculture, aquaculture and pharmaceutical manufacturing. FEMS Microbiol. Ecol..

[4] Nappier SP, Liguori K, Ichida AM, Stewart JR, Jones KR (2020). Antibiotic resistance in recreational waters: State of the science. Int. J. Environ. Res. Public Health.

[5] Thornber K (2022). Antimicrobial resistance in aquaculture environments: unravelling the complexity and connectivity of the underlying societal drivers. Environ. Sci. Technol..

[6] Lulijwa R, Rupia EJ, Alfaro AC (2020). Antibiotic use in aquaculture, policies and regulation, health and environmental risks: a review of the top 15 major producers. Rev. Aquac..

[7] Schar D, Klein EY, Laxminarayan R, Gilbert M, Van Boeckel TP (2020). Global trends in antimicrobial use in aquaculture. Sci. Rep..

[8] Rico A (2012). Use of chemicals and biological products in Asian aquaculture and their potential environmental risks: A critical review. Rev. Aquac..

[9] Chen J (2020). Antibiotics and food safety in aquaculture. J. Agric. Food Chem..

[10] Chen J, Wang Y, Chen X, Hesketh T (2020). Widespread illegal sales of antibiotics in Chinese pharmacies—A nationwide cross-sectional study. Antimicrob. Resist. Infect. Control.

[11] Price D, Sanchez J, Ibarra R, St-Hilaire S (2019). Variation in the concentration of antibiotics in tissue during oral antibiotic treatments in farmed salmonid. Aquaculture.

[12] Rigos G, Alexis M, Nengas I (1999). Leaching, palatability and digestibility of oxytetracycline and oxolinic acid included in diets fed to seabass *Dicentrarchus labrax* L.. Aquac. Res..

[13] Barreto FM (2018). Evaluation of the leaching of florfenicol from coated medicated fish feed into water. Environ. Pollut..

[14] Leung KC (2020). Fraudulent antibiotic products on the market for aquaculture use. Prev. Vet. Med..

[15] Andersson D, Hughes D (2014). Microbiological effects of sublethal levels of antibiotics. Nat. Rev. Microbiol..

[16] Liu B, Zhang X, Ding X, Wang Y, Zhu G (2021). Regulatory mechanisms of sub-inhibitory levels antibiotics agent in bacterial virulence. Appl. Microbiol. Biotechnol..

[17] Hasan, M. R., Bueno, P. B. & Corner, R.A. (eds). Strengthening, empowering and sustaining small-scale aquaculture farmers’ associations. FAO Fisheries and Aquaculture Technical Paper No. 655. Rome, FAO. 190. 10.4060/c7741en (2020).

[18] Agriculture, Fisheries and Conservation Department (AFCD). Marine fish culture, pond fish culture and oyster culture. https://www.afcd.gov.hk/english/fisheries/fish_aqu/fish_aqu_mpo/fish_aqu_mpo.html (2022).

[19] Theel ES, Schmitt BH, Cunningham SA, Walchak RC, Patel R, Wenegenack NL (2012). Formic acid-based direct, on-plate testing of yeast and *Corynebacterium* species by Bruker Biotyper matrix-assisted laser desorption ionization-time of flight mass spectrometry. J. Clin. Microbiol..

[20] Bauer AW, Kirby WMM, Sherris JC, Turck M (1966). Antibiotic susceptibility testing by a standardized disk method. Amer. J. Clin. Path..

[21] Clinical and Laboratory Standards Institute (CLSI). Performance standards for antimicrobial disk susceptibility tests, 13th ed. https://clsi.org/media/1925/m02ed13_sample.pdf (2018).

[22] Whitman KA, MacNair NG (2003). Finfish and Shellfish Bacteriology Manual Techniques and Procedures.

[23] Centre for Health Protection (CHP). Antimicrobial use surveillance in human – public sector. *Statistics on antimicrobial resistance control*. https://www.chp.gov.hk/en/static/101607.html (2021).

[24] Aquafeed. Suppliers’ news. *New label for AQUAFLOR offers more options, flexibility to U.S. fish farmers*. https://www.aquafeed.com/products/suppliers-news/new-label-for-aquaflor-offers-more-options-flexibility-to-us-fish-farmers/ (2012).

[25] Syndel. Terramycin® 200 for fish (oxytetracycline). https://syndel.com/product/terramycin-200-for-fish-oxytetracycline/ (2019).

[26] Risk Assessment Report Florfenicol. Food Safety Commission of Japan.FS/6/2016. http://www.fsc.go.jp/english/evaluationreports/amr_bacteria.data/kya20081030830_212.pdf (2016).

[27] World sea temperature. Hong Kong water temperature. https://www.seatemperature.org/asia/hong-kong/hong-kong.htm (2022).

[28] Yang F (2020). Effects of water temperature on tissue depletion of florfenicol and its metabolite florfenicol amine in crucian carp (*Carassius auratus gibelio*) following multiple oral doses. Aquaculture.

[29] Zanuzzo FS, Ellen de Fátima CP, Sandrelli RM, St-Hilaire S, O’Brien N, Gamperl AK (2022). Temperature has considerable effects on plasma and muscle antibiotic concentrations in Atlantic salmon (*Salmo salar*). Aquaculture.

[30] U.S. Environmental Protection Agency (USEPA). Method 1694: Pharmaceuticals and personal care products in water, soil, sediment, and biosolids by HPLC/MS/MS. Washington D.C. https://www.epa.gov/sites/default/files/2015-10/documents/method_1694_2007.pdf (2007).

[31] Alday-Sanz, V., Corsin, F., Irde, E. & Bondad-Reantaso, M. G. Survey on the use of veterinary medicines in aquaculture in *Improving biosecurity through prudent and responsible use of veterinary medicines in aquatic food production* (eds. Bondad-Reantaso, M. G., Arthur, J. R., & Subasinghe, R. P.) 29–44 (FAO Fisheries and Aquaculture Technical Paper No. 547, 2012). https://www.fao.org/3/ba0056e/ba0056e.pdf

[32] Bravo, S. Environmental impacts and management of veterinary medicines in aquaculture: the case of salmon aquaculture in Chile in *Improving biosecurity through prudent and responsible use of veterinary medicines in aquatic food production* (eds. Bondad-Reantaso, M. G., Arthur, J. R., & Subasinghe, R. P.) 11–24 (FAO Fisheries and Aquaculture Technical Paper No. 547, 2012). https://www.fao.org/3/ba0056e/ba0056e.pdf

[33] Drainage Service Department (DSD). List of sewage treatment facilities. https://www.dsd.gov.hk/EN/Sewerage/Sewage_Treatment_Facilities/List_of_Sewage_Treatment_Facilities/index.html (2019).

[34] Environmental Protection Department (EPD). Silverstrand beach. *Environmental protection interactive centre.* https://cd.epic.epd.gov.hk/EPICDI/beach/gradingreport/ss/ (2022).

[35] Archana A, Li L, Shuh-Ji K, Thibodeau B, Baker DM (2016). Variations in nitrate isotope composition of wastewater effluents by treatment type in Hong Kong. Mar. Pollut. Bull..

[36] Li D (2010). Antibiotic resistance characteristics of environmental bacteria from an oxytetracycline production wastewater treatment plant and the receiving river. Appl. Environ. Microbiol..

